# Severe and Persistent Thrombocytopenia in an Adolescent Girl Following Dengue Infection Manifesting As Pulmonary Hemorrhage

**DOI:** 10.7759/cureus.72127

**Published:** 2024-10-22

**Authors:** Ratan Kumar, Binod Kumar, Adyasha Mishra, Radhika Narayan, Vadde Sumitha

**Affiliations:** 1 Pediatrics, Manipal Tata Medical College, Jamshedpur, IND; 2 Pediatrics, Tata Main Hospital, Jamshedpur, IND; 3 Dermatology, Tata Main Hospital, Jamshedpur, IND; 4 Pathology, Tata Main Hospital, Jamshedpur, IND

**Keywords:** dengue, hemoptysis, immune thrombocytopenia, pulmonary hemorrhage, steroid

## Abstract

Thrombocytopenia is common in the critical phase of dengue due to decreased production from the bone marrow and increased peripheral destruction of platelets. Platelet count improves with resolution of the disease. However, further evaluation is required if thrombocytopenia persists for a longer period following dengue infection. Immune thrombocytopenic purpura (ITP) is an autoimmune hematological disorder in which antibodies bind to platelets, leading to accelerated platelet destruction, and is characterized by mucocutaneous bleeding. It may occur secondary to viral infections. We present a case of an adolescent girl with persistent severe thrombocytopenia following a dengue infection, which manifested as pulmonary hemorrhage. A bone marrow examination revealed a diagnosis of ITP, following which the child responded to steroid therapy.

## Introduction

Dengue is a mosquito-borne viral disease and has become endemic on all continents [1]. Thrombocytopenia is common in the critical phase of dengue, which improves with resolution of the disease [2]. However, if thrombocytopenia persists for a longer period following dengue infection, further evaluation is warranted.

Immune thrombocytopenic purpura (ITP) is a hematological disorder characterized by mucocutaneous bleeding. It is an autoimmune condition in which antibodies bind to platelets, leading to accelerated platelet destruction and clearance [3]. It can be primary or secondary. The secondary form of this disease may occur in various conditions like systemic lupus erythematosus (SLE), antiphospholipid antibody syndrome, immunodeficient states, lymphoproliferative disorders, viral infections, and drugs such as quinidine, sulfa drugs, and heparin [4]. Viral infections, such as human immunodeficiency virus (HIV), hepatitis C virus, varicella-zoster virus, rubella, influenza, Epstein-Barr virus, and parvovirus B19, have also been reported to precede ITP [5].

## Case presentation

A 14-year-old adolescent, previously healthy girl, completely immunized as per the national immunization schedule, with no significant past or family history, was admitted with complaints of petechial rashes all over the body for seven days, blood mixed vomiting for four days, and bleeding from gum for two days. There was a history of fever for two days, 10 days back. Rashes began on the third day of fever as petechiae and ecchymoses in the oral mucosa, gradually spreading to the upper and lower limbs, followed by the trunk, increasing in number and size over the days. On admission, she had tachycardia, pallor, and multiple ecchymosis over the forehead and left forearm with a purpuric rash over bilateral upper and lower limbs, and an active oral mucosal bleed. She was afebrile with stable vitals and systemic examination within normal limits. There was no lymphadenopathy or organomegaly.

Initial investigations revealed severe normocytic hypochromic anemia with anisocytosis (hemoglobin: 6.5 g%), severe thrombocytopenia (2000/mm3), neutrophilic leucocytosis (total leucocyte count: 23,600/mm3, neutrophil: 84%), indirect hyperbilirubinemia (total serum bilirubin: 1.91 mg/dl, direct serum bilirubin: 0.51 mg/dl), hyponatremia (Na+-133 mEq/L), and hypokalemia (k+-3.2 mEq/L). Dengue IgM ELISA was positive. Her blood and urine cultures were sterile. The chest X-ray on the day of admission was within normal limits. She was transfused with blood products, including packed red blood cells on day 2 and platelets daily for the first three days of hospital stay, in view of anemia and thrombocytopenia, refractory to blood products, and bleeding manifestations.

**Table 1:** Laboratory data Hb: hemoglobin, HCT: hematocrit, RBC: red blood cell, TLC: total leucocyte count, CRP: C-reactive protein, ALT: alanine transaminases, AST: aspartate transaminase, ALP: alkaline phosphatase, LDH: lactate dehydrogenase, PT: prothrombin time, INR: international normalized ratio, APTT: activated partial thromboplastin time

Laboratory investigations	Value (day of hospital stay)				Reference range (and unit)
	Day 1	Day 2	Day 3	Day 4	
Blood investigations					
Hb	6.5	7.1	6.2	6.9	11.5-16.5 g/dl
HCT	21.2	23.5	21.6	23	35-50 %
RBC Count	2.3	2.6	2.3	2.5	4.5-5.5 million/ mm^3^
TLC	23,600	16,000	15,100	15,100	4,000-11,000/mm^3^
Neutrophil	85	78	76	85	60-70%
Lymphocyte	12	13	15	11	30-40%
Platelet	2000	3000	3000	3000	150,000-410,000/mm^3^
CRP	0.51	-	-	-	0.08-0.79 mg/dl
Urea	71.4	-	-	-	18-45 mg/dl
Creatinine	0.65	-		-	0.5-1.5 mg/dl
Calcium	9.4	-	-	-	8.6-10.3 mg/dl
Sodium	133	-	139	-	136-146 mEq/l
Potassium	3.2	-	3.1	-	3.5-5.5 mEq/l
Chloride	98	-	105	-	95-108 mEq/l
Total bilirubin	1.91	-	-	-	0.2-1 mg/dl
Direct bilirubin	0.51	-	-	-	0.1-0.5mg/dl
ALT	28.7	-	-	-	0-45 U/L
AST	54.8	-	-	-	0-35 U/L
ALP	152.90	-	-	-	53-141 U/L
LDH	914.7	-	-	-	208-378 U/L
PT	17.4	-	-	-	10.3-16.5 sec
INR	1.31	-	-	-	0.8-1.2
APTT	21	-	-	-	23.4-38.4 sec
Reticulocyte count	9.31	-	-	-	-
Corrected reticulocyte count	4.38	-	-	-	0.5-2 %

**Figure 1:**
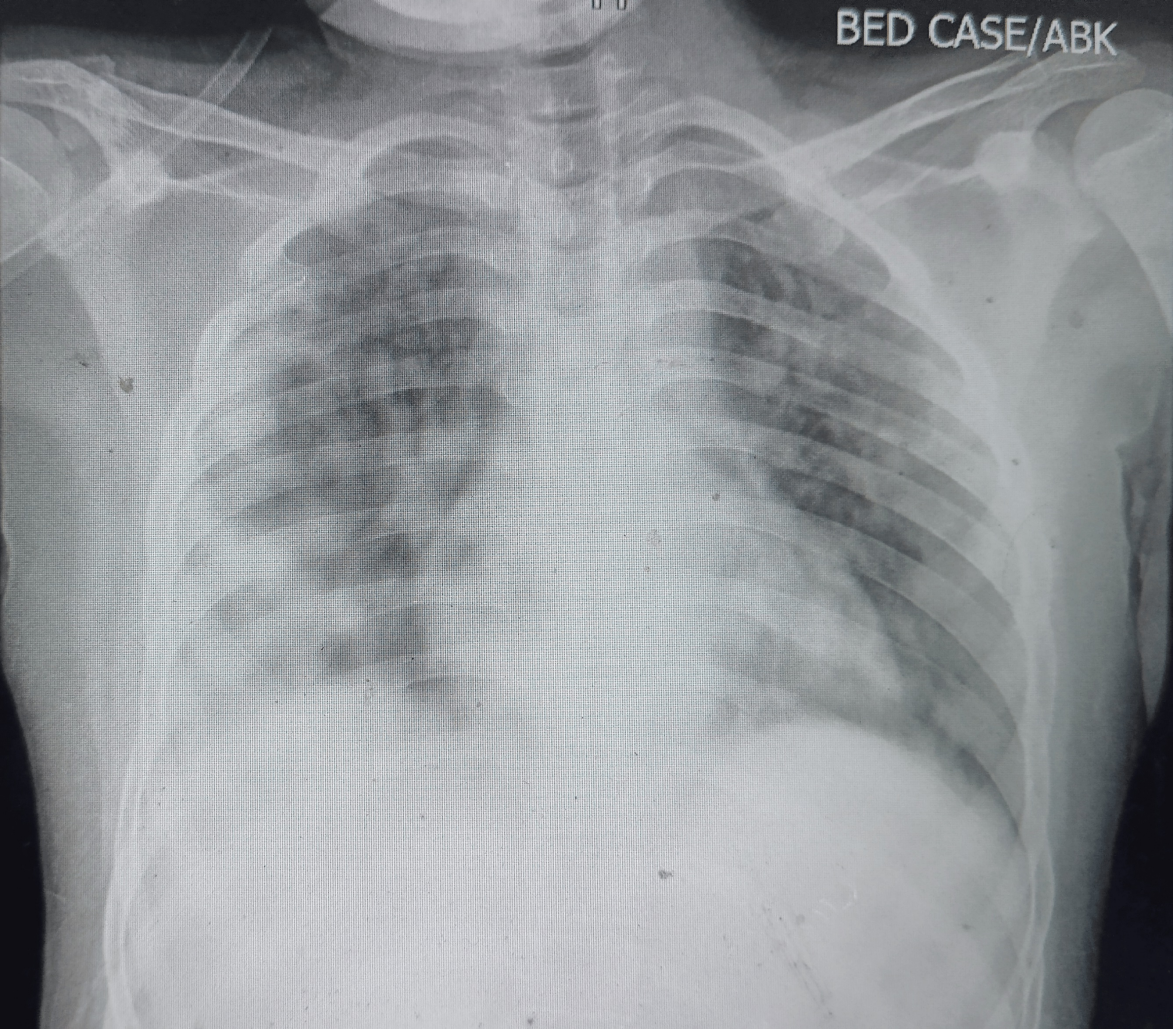
Chest X-ray on day 3 of hospitalization

Another workup for anemia showed raised LDH (914.7 mg/dl) with reticulocytosis (corrected reticulocyte count: 4.8%); however, the direct Coombs test was negative and there has been no previous history of blood transfusion. On the third day of her hospital stay, she developed hemoptysis, tachypnea, chest retractions, and crepitations on auscultation, along with desaturation. A chest X-ray (Figure 1) revealed bilateral, homogenous infiltrates. Signs, symptoms, and chest X-rays were suggestive of pulmonary hemorrhage. She was supported with non-invasive ventilation.

**Figure 2:**
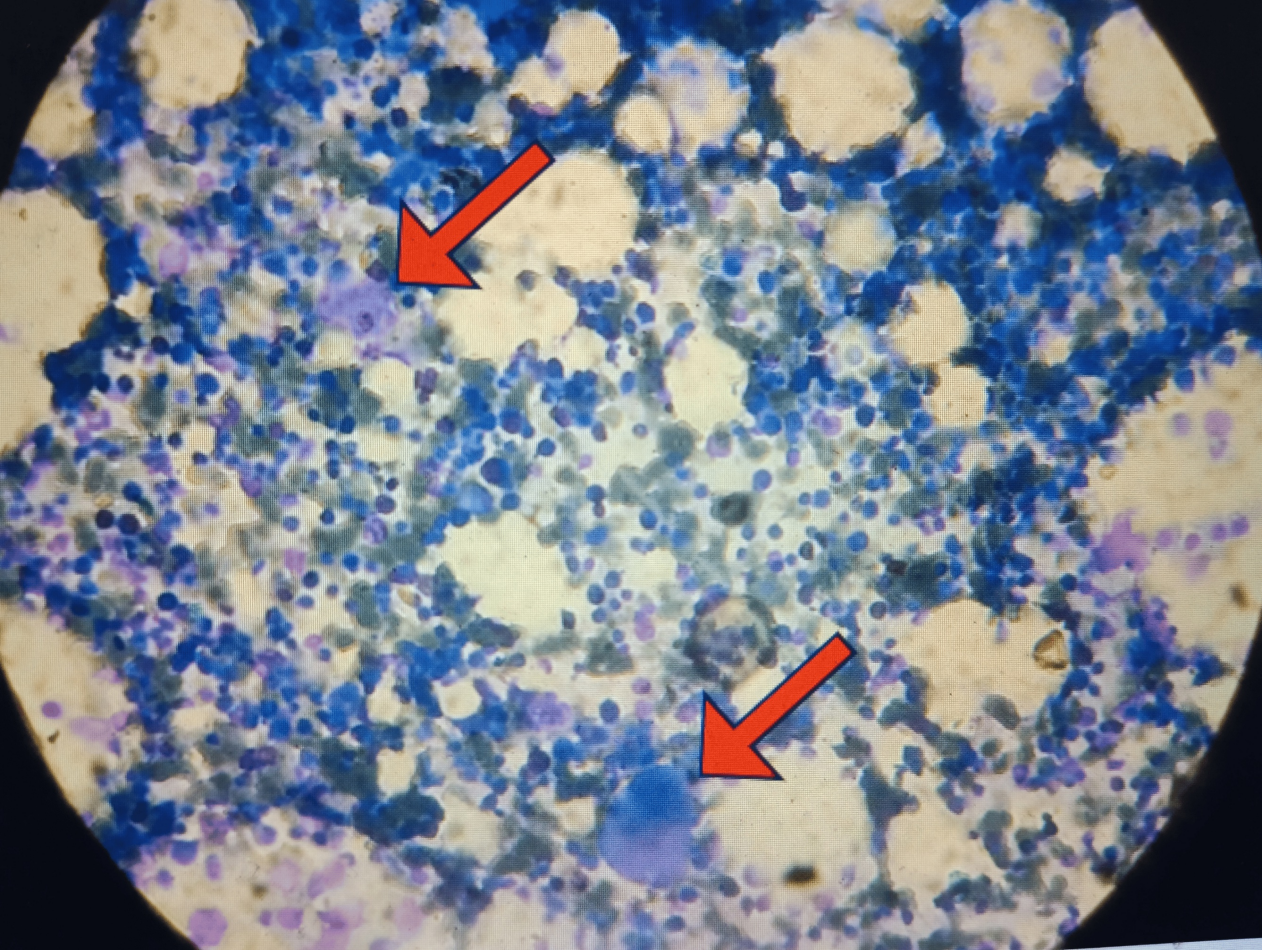
Bone marrow slide at 100x magnification showing megakaryocytic thrombocytopenia Red arrows: immature megakaryocytes. The megakaryocytes show hypolobation, hypogranulation, and decreased budding. Background: mild erythroid hyperplasia with a dimorphic erythroid series ranging from micronormoblastic to megaloblastic is observed.

Since two cell lines were depressed, bone marrow aspiration and biopsy were done, which showed mild erythroid hyperplasia with dimorphic micro-normoblastic to megaloblastic erythroid series. There were a fair number of immature megakaryocytes (Figure 2). There was no evidence of granuloma or malignancy. Therefore, bone marrow studies were favoring the diagnosis of ITP.

She was started on oral corticosteroid (prednisolone) at a dose of 4 mg/kg/day. Bleeding manifestations subsided; platelets started to increase from the third day of starting steroids. Hence, she was discharged with advice to continue prednisolone to complete a total of five days. On follow-up, her platelet count became normal, and she recovered well.

## Discussion

Viral infection causes thrombocytopenia by multiple mechanisms. It includes platelet consumption by inflammation-induced coagulation, phagocytosis, and decreased production due to cytokine-induced myelosuppression [6]. In dengue infection, thrombocytopenia is mainly attributed to a decrease in bone marrow production and/or increased peripheral destruction and immune-mediated clearance of platelets [7,8].

ITP has an acute course in children following viral infection, while in adults it commonly follows a chronic course [5]. Although dengue fever has been reported to cause chronic ITP even in children [9-11]. ITP commonly causes mucocutaneous bleeding manifesting as petechiae and epistaxis but can also cause potentially life-threatening gastrointestinal, intracranial, or urinary tract hemorrhage [12]. Lethal alveolar hemorrhage in cases of ITP has also been reported in the literature [13,14]. Steroid is the first-line treatment in cases of ITP secondary to viral infection. Persistent thrombocytopenia unresponsive to steroids has been reported to respond to intravenous immunoglobulin [15].

The differential diagnosis for our case can be Evans syndrome, which is an autoimmune condition that presents with two or more cytopenias, commonly including autoimmune hemolytic anemia and ITP, with or without immune neutropenia. It can be primary or secondary to conditions like SLE, common variable immunodeficiency, and autoimmune lymphoproliferative syndrome in non-Hodgkin lymphoma in older patients, chronic lymphocytic leukemia, viral infections (such as HIV, hepatitis C), and following allogeneic hematopoietic cell transplantation [16]. The points in favor of this diagnosis include female gender and severe thrombocytopenia leading to life-threatening hemorrhage. The points against Evans syndrome include lack of splenomegaly, negative direct Coombs test, and no relapse of bicytopenia with a short course of steroids.

## Conclusions

Secondary immune thrombocytopenia should be suspected if a case of dengue fever presents with persistent thrombocytopenia. Dengue-induced severe thrombocytopenia can present as pulmonary hemorrhage, which should urgently be identified and treated with steroids or other immunomodulators.
